# Supplementary material for: Description of Gut Mycobiota Composition and Diversity of Caprinae Animals
Source: Microbiol Spectr. 2023 Jan 10;11(1):e02424-22. doi: 10.1128/spectrum.02424-22 (PMC9927506; doi:10.1128/spectrum.02424-22)
Supplement: Supplemental file 2 — Fig. S1 and S2. Download spectrum.02424-22-s0002.pdf, PDF file, 3.7 MB [file spectrum.02424-22-s0002.pdf]

Supplemental Figure 1

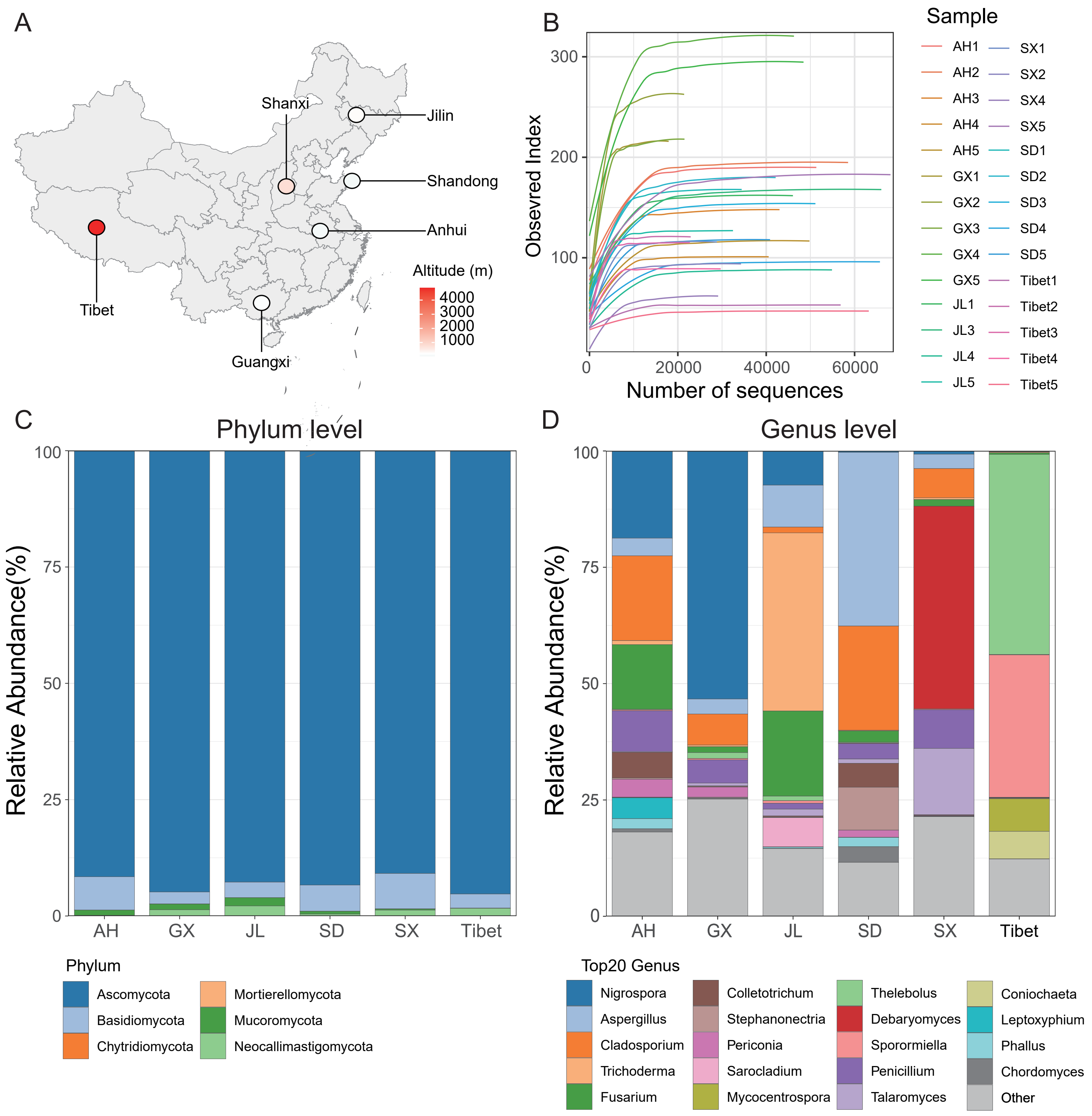

Supplementary Fig. 1:Composition of gut mycobiome from difference provinces. (A) The geographical diagram of the sampling sites, with the gradient in red representing the altitude of the sampling sites.(B) The rarefaction curves showing that the relationship between sequencing depth and Observed ASVs. (C, D) Community composition of the gut mycobiota in six provinces at the phylum and genus levels, respectively.

Supplementary Figure 2

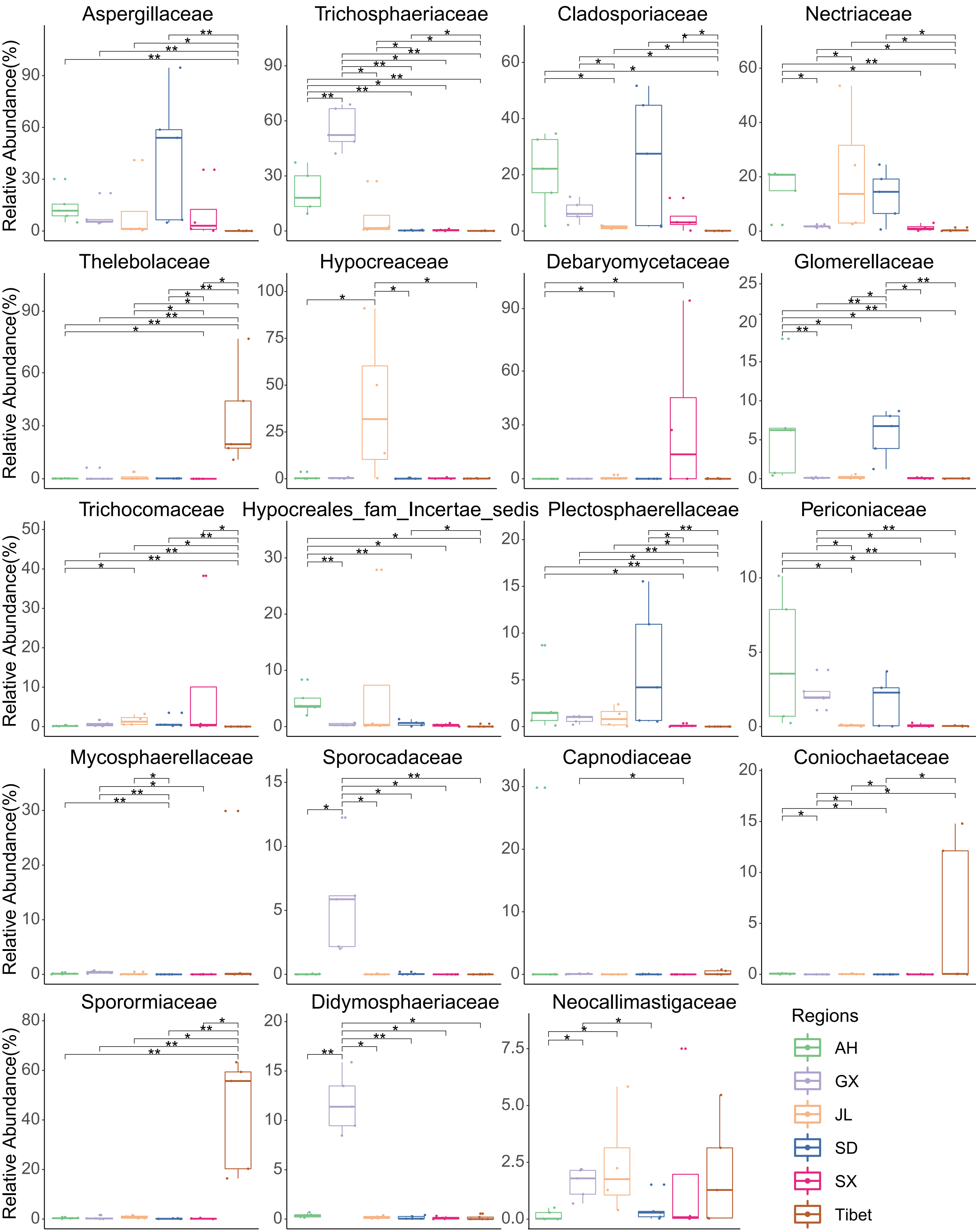

Supplementary Fig. 2: Boxplots show differences in the abundance of top 20 family. Asterisks indicate a statistical significance (Wilcoxon rank sum): *, $p < 0.05$; **, $p < 0.01$.
